# Improved Hydrogen Separation Using Hybrid Membrane Composed of Nanodiamonds and P84 Copolyimide

**DOI:** 10.3390/polym10080828

**Published:** 2018-07-27

**Authors:** Alexandra Pulyalina, Galina Polotskaya, Valeriia Rostovtseva, Zbynek Pientka, Alexander Toikka

**Affiliations:** 1 Institute of Chemistry, Saint Petersburg State University, Universitetskiy pr. 26, 198504 Saint Petersburg, Russia; g_polotskaya@mail.ru (G.P.); st017536@student.spbu.ru (V.R.); a.toikka@spbu.ru (A.T.); 2 Institute of Macromolecular Compounds, Russian Academy of Sciences, Bolshoy pr. 31, 199004 Saint Petersburg, Russia; 3 Institute of Macromolecular Chemistry, Czech Academy of Sciences, Heyrovsky Sq. 2, 16206 Prague, Czech Republic; pientka@imc.cas.cz

**Keywords:** membrane, gas separation, nanomodifiers, hydrogen separation, methane steam reforming

## Abstract

Membrane gas separation is a prospective technology for hydrogen separation from various refinery and petrochemical process streams. To improve efficiency of gas separation, a novel hybrid membrane consisting of nanodiamonds and P84 copolyimide is developed. The particularities of the hybrid membrane structure, physicochemical, and gas transport properties were studied by comparison with that of pure P84 membrane. The gas permeability of H_2_, CO_2_, and CH_4_ through the hybrid membrane is lower than through the unmodified membrane, whereas ideal selectivity in separation of H_2_/CO_2_, H_2_/CH_4_, and CO_2_/CH_4_ gas pairs is higher for the hybrid membrane. Correlation analysis of diffusion and solubility coefficients confirms the reliability of the gas permeability results. The position of P84/ND membrane is among the most selective membranes on the Robeson diagram for H_2_/CH_4_ gas pair.

## 1. Introduction

Membrane technologies provide a number of advantages in terms of environmental requirements, high energy efficiency, and low capital and operating cost over their conventional counterpart technologies [1]. Membrane gas separation has been successfully developed and is widely used in chemical and petrochemical plants. Separation of hydrogen from its mixtures with nitrogen or hydrocarbons, nitrogen purification, and carbon dioxide removal from natural gas are recognized as the most significant industrial applications of membranes [2].

Hydrogen separation and purification is among the global problems; hydrogen is considered to be the most promising source of alternative energy that could replace fossil fuel. Hydrogen is a “green” fuel, since the only product of its combustion is water, which does not damage the environment [3]. Hydrogen is commonly produced by steam methane reforming [4]:

CH_4_ + H_2_O ↔ CO + 3H_2_

CO + H_2_O ↔ CO_2_ + H_2_

Hydrogen is purified to satisfy the various purity requirements for different applications [5]: high purity < 99.99% is required in fuel cells, ~70–80% purity is sufficient for hydrocracking. Hydrogen can be enriched by various methods such as pressure-induced adsorption [6], cryogenic distillation [7], and membrane separation [8].

Membrane gas separation is a promising technology for hydrogen purification [9]. The process of selective separation is based on the different permeability of membranes for individual components of the gas mixture. The search for new membrane materials with improved transport properties is one of the priority scientific tasks. Among various membrane polymers, aromatic polyimides and their derivatives are of undoubted interest due to their unique gas separation properties, which are combined with high chemical and thermal stability [10,11,12]. The problem of the hydrogen separation from CO_2_ and CH_4_ has been studied using membranes based on aromatic polyimides [13,14], their mixtures with polymers of another nature [15], and the products of polyimide chemical modification with cross-linking agents [16,17,18,19,20].

It has recently been found that gas separation properties can be improved by development of hybrid membranes consisting of nanoparticles dispersed in a polymer matrix: zeolites [21], carbon molecular sieves [22], carbon nanotubes [23], graphene [24], others [25] or nanoparticles of a different nature [26,27,28].

Nanodiamonds (ND) or ultradispersed diamonds are specific carbon particles produced by detonation synthesis using explosive mixtures [29,30]. Subsequent chemical purification of ND using strong oxidizing agents (sulfuric acid, oleum, nitric acid, etc.) completes the formation of the ND particles. ND is a compound particle with a three-layer structure including (i) a diamond core (4–6 nm), in which up to 70–90 wt% C atoms are located; (ii) a transition carbon shell, an intermediate X-ray amorphous layer (0.4–1.0 nm), where 10–30% C atoms are located; and (iii) a surface layer containing (in addition to C) N, O, H atoms forming functional groups. ND particles attract special attention due to the chemical stability of their diamond core and the surface activity given by various functional groups (‒OH, ‒COOH, =C=O, etc.) that appear during the chemical purification of detonation ND using strong acids. ND have already found application as a component of mixed matrix membranes for the solving of different industrial problems. Nanocomposite membranes based on poly(vinylidene fluoride) modified with ND have been applied to water desalination by distillation [31]. The polyphenylene-*iso*-phthalamide membrane containing ND in the matrix has been successfully used in pervaporation [32] and gas separation [33]. ND particles have been selected as an inorganic modifier of poly(phenylene oxide) membranes for gas separation [34].

**Figure 1:**
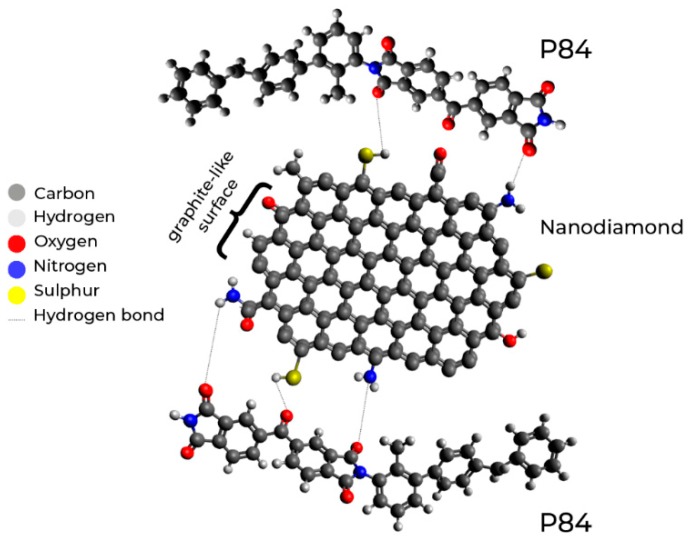
Structure of nanodiamonds and P84 copolyimide.

In the present work, ND were chosen as the filler of the P84 copolyimide matrix to develop novel hybrid membrane. Figure 1 shows the structure of P84 copolyimide and nanodiamonds. The P84 copolyimide {BTDA-TDI/MDI, co-polyimide of 3,3′,4,4′-benzophenone tetracarboxylic dianhydride (BTDA) and 80% toluene diisocyanate (TDI) + 20% methylene diphenyl diisocyanate (MDI)} is a commercially available polymer that demonstrates good mechanical properties, chemical resistance, and low hydrophilicity; it has already been studied as a membrane material for ultrafiltration [35], nanofiltration [36], gas separation [37], and pervaporation [38].

The aim of the present work is to obtain the hybrid membrane based on P84 copolyimide modified with ND particles, to study its structure, physicochemical and gas transport properties in hydrogen separation from components of steam methane reforming.

## 2. Materials and Methods

### 2.1. Materials

The P84 copolyimide was purchased from HP Polymer GmbH (Lenzing, Austria). Nanodiamonds with density 3.0 g/cm^3^ were produced by detonation synthesis and provided by SCTB ‘Technolog’ (Saint Petersburg, Russia). *N*,*N*-Dimethylacetamide (DMA) manufactured by Vekton (Saint Petersburg, Russia) was used as solvent without further purification.

### 2.2. P84/ND Composite Preparation

The P84/ND composite was obtained by thoroughly mixing powders of 99 wt% P84 and 1 wt% ND in agate mortar for 1 h. Thereafter, the composite powder was dissolved in DMA to obtain a solid phase concentration of 8 wt%. To achieve complete homogenization of the composite solution, intensive stirring with a mechanical stirrer for 1 h and then ultrasonic bath at 40 °C for 40 min were used. After that, the P84/ND composite solution was filtered to remove any mechanical impurities. 

### 2.3. Membrane Formation

Dense membranes ~30 µm thick were prepared by casting the 8 wt% polymer solution in DMA onto a glass plate. The solvent was removed by evaporation at 40 °C for 48 h; the membranes were separated from the support and dried in a vacuum oven at 60 °C for about 2 weeks in order to achieve constant weight.

### 2.4. Characterization of P84 and P84/ND

A multimode Atomic Force Microscope Nanoscope IIIa (Digital Instruments, Santa Barbara, CA, USA) was used to observe the nanoparticles in situ in tapping mode using OTESPA silicon cantilevers (Veeco Instruments, Dourdan, France) with a radius of 5 nm and oscillating at 300 kHz.

For investigating the membrane cross-sectional morphology, the membranes were cracked in liquid nitrogen, coated with carbon, and observed using Zeiss Merlin scanning electron microscope (Zeiss AG, Oberkochen, Germany).

The membrane density, *ρ*, was estimated by the flotation method in a solution of isopropanol-carbon tetrachloride at 25 °C [*ρ*(*i*PrOH) = 0.786 g/cm^3^, *ρ*(СCl_4_) = 1.594 g/cm^3^]. 

Fractional free volume of P84 membrane, *FFV*, was calculated by the Bondy method [39]:

(1) $${FFV=(V}_{0}-1{.3V}_{w}{)/V}_{0}$$

where *V*_0_ = 1/*ρ_P84_* is the polymer specific volume and *V_w_* is the van der Waals volume of P84 estimated via Askadskii’s group contribution method.

Fractional free volume of P84/ND composite, *FFV_c_*, was calculated by the following equation [40]:

(2) $$FFVc=1-\rho_{c}(1-\frac{wt_{ND}\%}{100})\cdot (1-\mathrm{FFV}){\rho_{P84}}^{-1}-\rho_{C}\rho_{ND}^{-1}\frac{wt_{ND}\%}{100}$$

where *ρ_P84_*, *ρ_ND_*, and *ρ_C_* are the densities of polymer, nanodiamonds, and composites, respectively, and wt_ND_ is the weight fraction of the ND modifier in the polymer matrix.

Water contact angles of investigated membranes were measured by the sessile drop method on the Drop Shape Analyzer DSA 10 (KRÜSS GmbH, Hamburg, Germany) at 20 °C and atmospheric pressure.

Thermogravimetric analysis (TGA) was carried out using samples of 8–15 mg at a heating speed of 10 °C/min in a nitrogen atmosphere. A TG 209 F3 Iris thermo-microbalance (Netzsch, Selb, Germany) was used for the analysis.

### 2.5. Gas Permeation Measurement

Gas permeability of membranes was measured using single gases with high purity (H_2_, CO_2_, CH_4_) by the barometric technique using a laboratory high-vacuum apparatus with a static permeation cell with an effective area of 5.25 cm^2^ at 30 °C. The membrane sample was placed and sealed in a module which was evacuated. At the beginning of the permeation experiment, the gas under constant pressure, *p* (150 kPa), was brought into the feed part of the permeation cell. The permeability was determined from the increase of pressure *Δp_p_* in a calibrated volume *V_p_* of the product part of the cell per the time *Δt* interval during steady-state permeation. The gas permeability coefficient, *P_exp_*, was estimated by the following equation [41]:

(3) $$P_{\exp}=\frac{\Delta p_{p}}{\Delta t}\cdot \frac{V_{p}\cdot l}{S\cdot p}\cdot \frac{1}{RT}$$

where *l* is a membrane thickness, *S* is its area, *T* is the absolute temperature, and *R* is the gas constant. The permeability coefficient P was expressed in Barrers (1 Barrer = 10^−10^ cm^3^ (STP)cm/(cm^2^ s cmHg)). Each experiment was repeated 3–5 times; several membranes of approximately the same thickness and prepared under the same conditions were used. The relative error of permeability value was 1–3%.

The ideal selectivity of gas *i* relatively gas *j*, *α*_*i/j*_, was calculated with the accuracy ± 0.05 according to the following equation:

(4) $$\alpha_{i/j}=\frac{P_{i}}{P_{j}}$$

The diffusion coefficient, *D*, was calculated from the initial transient regime, which determines the x-intercept that is a time-lag, *θ*:

(5) $$D=\frac{l^{2}}{6\theta }$$

The solubility coefficient, *S*, was calculated using the main gas transport equation:

*P* = *S*·*D* (6)

Correlation analysis of gas transport parameters was performed using the data in Table 1.

The gas permeability coefficient for P84/ND film prepared by inclusion of non-porous impermeable filler in a continuous polymer matrix, *P_Maxwell_*, was calculated using Maxwell model [43]:

(7) $$P_{Maxwell}=P_{P84}\left(\frac{1-\varphi_{ND}}{1+\frac{\varphi_{ND}}{2}}\right)$$

where *P_P84_* is the gas permeability coefficient for pure polymer and *ϕ_ND_* is the volume fraction of nanodiamonds.

The volume fraction of the filler in the polymer matrix was estimated using the equation:

(8) $$\varphi_{ND}=\frac{wt_{ND}\%}{wt_{ND}\%+\frac{\rho_{ND}}{\rho_{P84}}(1-w_{ND})}$$

where *ρ_P84_* and *ρ_ND_* are the density of P84 and ND, respectively, and *wt_ND_* is the weight fraction of the filler in the polymer matrix. 

## 3. Results

Hybrid P84/ND membrane was obtained by dispersing 1 wt% ND particles in a matrix of P84 copolyimide. The certainty that all the calculated amount of ND used for the composite preparation is present in the membrane is guaranteed by the method of the composite preparation and subsequent hybrid membrane formation. To reveal specific features of the hybrid membrane, comparative research of P84/ND and pure P84 membranes on structure, physical and transport properties was performed.

### 3.1. Membrane Structure

**Figure 2:**
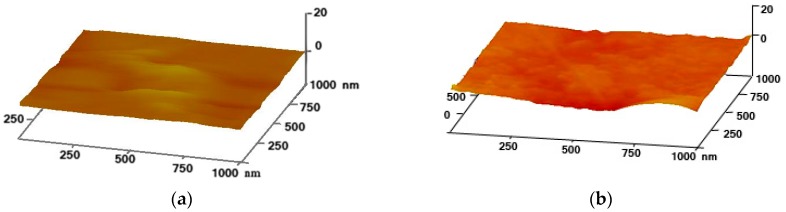
AFM images of membrane surfaces: (**a**) P84 and (**b**) P84/ND.

The change of membrane structure with inclusion of ND in the P84 matrix was studied by SEM and AFM. As seen from the AFM images of the membrane surfaces in Figure 2, the inclusion of ND nanoparticles in the membrane leads to a more complex structure in comparison to the smooth surface of pure P84. Namely, spherical fragments are formed on the surface of P84/ND membrane due to the aggregation of macromolecules in solution upon the addition of nanoparticles. Such a relief of the modified membrane can be formed as a result of the spontaneous movement of ND and aggregates to the surface of the hybrid membrane during the solvent evaporation.

**Figure 3:**
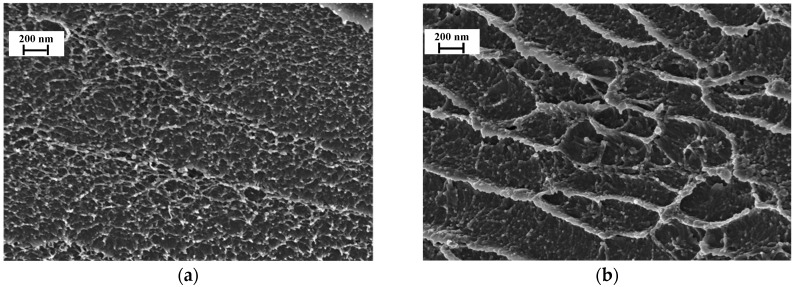
SEM micrographs of the membrane cross-section: (**a**) P84, (**b**) P84/ND.

SEM was used to study the morphology of membrane cross-sections (Figure 3). The cross-section of unmodified P84 membrane (Figure 3a) is homogenous and uniform. The structure of the hybrid P84/ND membrane is more complicated. The fracture lines can be observed across the entire micrograph with the inclusion of ND in P84 matrix. This observation would typically be an indication of reduced flexibility in the membrane. This fact can probably be explained by the aggregation of macromolecules and the formation of a denser structure of the P84/ND membrane. The inclusion of ND leads to less flexibility in the hybrid membrane. However, the absence of visible defects in the P84/ND cross-section indicates the good compatibility of the nanoparticles with P84 and the stability of the hybrid membrane [25].

### 3.2. Physical Properties

Table 2 lists some physical properties of the membranes under study: water contact angle, density, fractional free volume (*FFV*) and glass transition temperature (*T_g_*). The water contact angle on the surface of P84/ND membrane is decreased compared to the P84 membrane; this points to the moderate hydrophilization of the membrane surface due to modification with nanoparticles.

According to Table 2, the membrane density increases when the ND particles are incorporated into the polymer matrix. Fractional free volume of P84/ND membrane is decreased compared with the pure P84. These facts indicate that the membrane structure becomes more compact after inclusion of the nanomodifier into the polymer matrix. Only a very weak increase of T_g_ value is registered as a result of the inclusion of ND into P84 matrix.

### 3.3. Transport Properties

The selective separation of H_2_/CH_4_ and H_2_/CO_2_ is an important task due to the promising role of hydrogen as an energy source. The CO_2_/CH_4_ separation is an urgent problem, since the CO_2_ presence obstructs the development of oil and gas fields. Thus, industrial problems promote research on the transport properties of P84 and P84/ND membranes for the following gases: H_2_, CO_2_, and CH_4_. Table 3 lists gas permeability and ideal selectivity in the separation of the industrially significant gas pairs. It was found that the gas permeability coefficients of the P84/ND membrane are lower than those of pure P84. This fact is determined by the decreasing *FFV* values of membrane after inclusion of ND modifier (Table 2), because the permeability depends directly on the *FFV* value of the membrane [1].

Particular attention was paid to the ideal selectivity in the separation of three gas pairs: H_2_/CO_2_, H_2_/CH_4,_ and CO_2_/CH_4_. Our glassy polymer membranes were selectively permeable to H_2_, which can be explained by the diffusion selectivity due to the smaller size of H_2_ molecules. In the case of the CO_2_/CH_4_ pair, both diffusion and sorption factors facilitate CO_2_ selective permeability. Table 3 shows that values of the ideal selectivity of the P84/ND membrane are greater than those of pure P84 for all gas pairs. ND filler containing amino groups improves the interaction of the polymer matrix and the dispersed phase; this may lead to the rigid structure at the interface, which decreases the gas permeability but increases H_2_/CO_2_, H_2_/CH_4_, and CO_2_/CH_4_ ideal selectivity.

To study the influence of the composition and structure changes in membranes based on P84 copolyimide on gas transport properties, analysis of diffusion and solubility coefficients is required, because they are components of the permeability coefficient according to Equation (6). Data on diffusion and solubility coefficients are presented in Table 4.

With an increase of the gas molecule size (Table 1) the diffusion coefficients decrease. This is also reflected to the lowering of the gas permeability (Table 3). The decrease in both diffusion and sorption coefficients with the introduction of ND is probably connected with the strong interaction between nanoparticles and P84 and the formation of a denser structure around the modifiers. This fact will be discussed in more detail below

**Figure 4:**
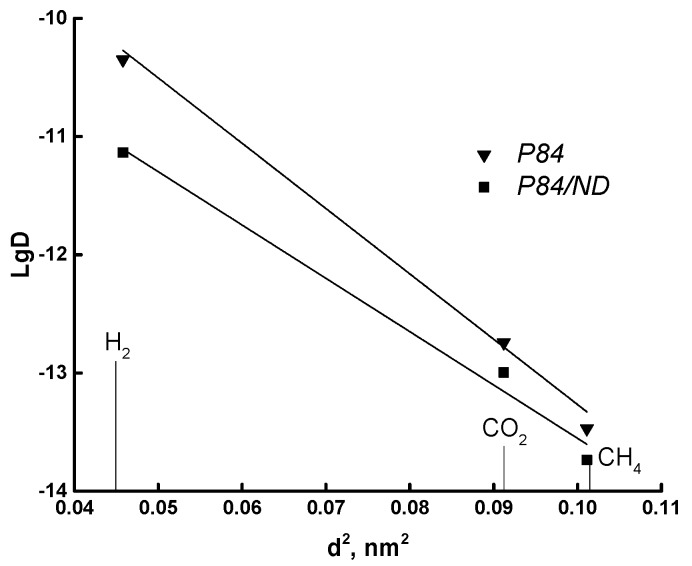
Dependence of diffusion coefficient on effective diameter of gas molecules for P84 and P84/ND membranes.

Correlation analysis of the membrane transport parameters was carried out using the Teplyakov technique [42]. The first correlation for the polymer–gas system is the dependence of the gas diffusion coefficient on the effective diameter of gas molecules. Figure 4 shows this dependence in the logarithmic coordinates lg*D* = *f* (*d*^2^) for both membranes. The diffusion coefficients follow a linear correlation with gas molecule diameters, and there is no discernible difference between the trends for P84 and P84/ND membranes. This indicates that the diffusion during gas separation occurs mainly due to elements of the free volume.

**Figure 5:**
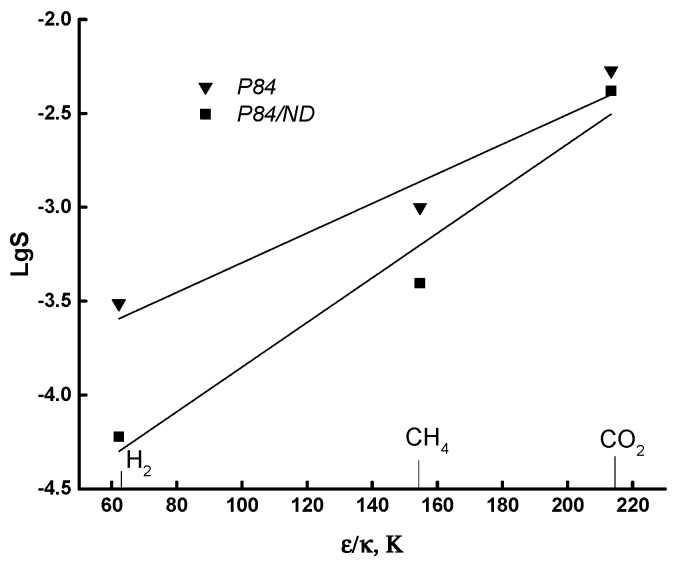
Dependence of solubility coefficients on the depth of the Lennard-Jones potential of gases for P84 and P84/ND membranes.

Another kind of correlation for the polymer–gas system is the dependence of the solubility coefficient on the depth of the Lennard-Jones potential (*ε*/*k*) of gases [42]. Figure 5 shows the dependence in logarithmic coordinates lg*S* = f (*ε*/*k*) for P84 and P84/ND membranes. The linear type of this correlation for both membranes confirms the reliability of the experimental results.

**Figure 6:**
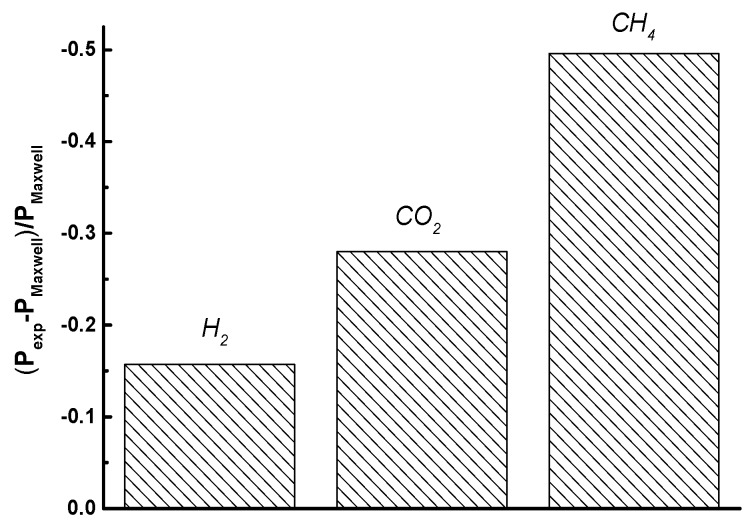
Relative deviation of gas permeability coefficients *P_exp_* from *P_Maxwell_* for the P84/ND membrane.

Numerous models have been developed to describe the effect of modifiers on permeability in gas separation [44,45,46,47]. One of the most widely known is Maxwell’s model, which was used to calculate permeability coefficients of H_2_, CO_2_, and CH_4_ for the P84/ND membrane and to estimate the deviation of experimental permeability coefficients *P_exp_* from *P_Maxwell_*. Figure 6 shows the relative permeability deviation for the P84/ND membrane, which is located in the negative region, since the experimental permeability of all gases is less than the calculated one. The reason is apparently associated with the stronger interaction of nanoparticles with a polymer matrix, which was not accounted for in the model.

It should be noted that the transport properties of hybrid membranes containing nanoparticles depend directly on their morphology. Gas permeability coefficients of hybrid membranes, where the interaction between matrix and particles is absent, can be predicted by the Maxwell model. However, an inadequate description of P84/ND permeability data by this model indicates the dependence of the decrease in permeability on changes in the membrane morphology.

**Figure 7:**
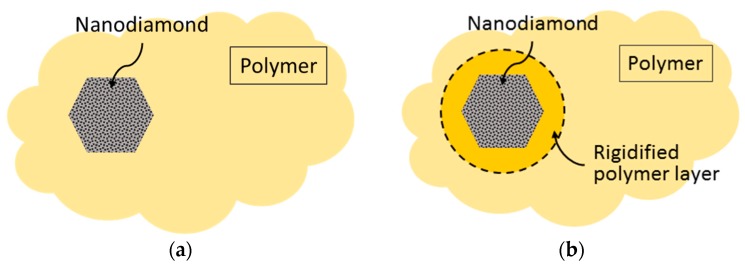
A schematic morphology of polymer membrane with dispersed nanoparticles: (**a**) ideal structure; (**b**) with rigidified polymer layer.

In general, formation of an ideal structure is very difficult because of the different physicochemical nature of two phases and their tendency towards aggregation. A decrease in the permeability of the P84/ND membrane can probably be explained by a decrease in the mobility of polymer chains due to an increase in the packing density around the nanoparticle [48,49]. Figure 7 shows the scheme of the ideal structure of the hybrid membrane (a) and the structure with a dense polymer layer around the nanoparticle (b).

**Figure 8:**
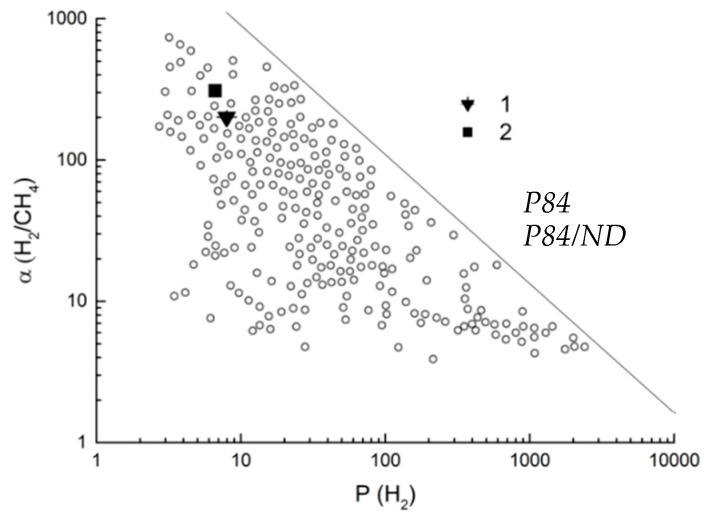
Robeson diagram for hydrogen/methane gas pair [50].

For an objective evaluation of the efficiency of the membranes under study, their gas transport properties were plotted in a Robeson diagram [50]. Figure 8 shows the Robeson diagram for the Н_2_/СН_4_ gas pair, i.e., the dependence of the ideal selectivity *α_Н2/СН4_* on the permeability coefficient *P_Н2_*. The straight line in Figure 8 represents the upper bound of the gas separation capabilities of all membranes known from scientific literature up to 2008 [50]. As can be seen from Figure 8, the P84 membrane is located among the most selective membranes, and the P84/ND hybrid membrane is displaced to a more advantageous position. This fact shows a promising means of membrane modification by nanodiamonds.

The transport properties of the P84/ND membrane were also compared with the latest literature data on the gas separation properties of modified polyimide membranes for the cases of H_2_/CO_2_, H_2_/CH_4_ and CO_2_/CH_4_ separation (Table 5). 

It can be seen that ideal selectivity of P84/ND exceeds this parameter for other presented membranes, but the permeability is noticeably lower. This can be explained by the difference in membrane topology. A promising way to improve permeability for industrial applications is the creation of a bilayer composite membrane with a thin~P84/ND selective layer.

## 4. Conclusions

A novel hybrid membrane was obtained by dispersing ND particles in a matrix of P84 copolyimide and was characterized by high selectivity in separation of hydrogen from gases formed during steam reforming of methane: H_2_, CO_2_, and CH_4_. The introduction of ND particles into the P84 matrix leads to a more compact macromolecular packing in the membrane. As a result, the experimental permeability of all gases through the P84/ND membrane is lower than the permeability calculated by the Maxwell model; this is due to stronger interaction of the nanoparticles with the polymer matrix than that model provided. Transport properties of membranes were studied for the following gases: H_2_, CO_2_, and CH_4_. Gas permeability coefficients of the hybrid P84/ND membrane are lower than that of the unmodified P84 membrane, whereas ideal selectivity in separation of H_2_/CO_2_, H_2_/CH_4_, and CO_2_/CH_4_ is higher for the hybrid membrane. Analysis of transport properties using correlation dependencies for diffusion and solubility coefficients of P84 and P84/ND membranes confirms the reliability of the gas permeability measurements and indicates that diffusion during gas separation occurs mainly due to elements of free volume.

## Figures and Tables

**Table 1:** Effective diameter, *d*, and the depth of the Lennard-Jones potential, (*ε*/*k*), for the gases under study [42].

Gas	d, nm	(*ε*/*k*), K
H_2_	0.210	62.2
CO_2_	0.302	213.4
CH_4_	0.318	154.7

**Table 2:** Physical properties of membranes.

Membrane	Contact Angle of Water, °	Density, g/cm^3^	Fractional Free Volume	T_g_, °C
P84	72.0 ± 0.5	1.323 ± 0.005	0.082 ± 0.01	344 ± 3
P84/ND	70.0 ± 0.4	1.343 ± 0.003	0.073 ± 0.01	346 ± 3

**Table 3:** Transport properties of membranes, 30 °С.

Membrane	Permeability, Barrer			Ideal Selectivity		
	H_2_	CO_2_	CH_4_	H_2_/CO_2_	H_2_/CH_4_	CO_2_/CH_4_
P84	8.0	2.25	0.040	3.6	200	56
P84/ND	6.7	1.61	0.022	4.1	310	75

**Table 4:** Diffusion coefficients and solubility coefficients for H_2_, CO_2_, and CH_4_.

Membrane	Diffusion Coefficient, D × 10^−12^ m/s			Solubility Coefficients, S × 10^−3^ mol/(m^3^ Pa)		
	H_2_	CO_2_	CH_4_	H_2_	CO_2_	CH_4_
P84	44.7	0.18	0.034	0.31	5.0	1.0
P84/ND	7.3	0.10	0.018	0.06	4.0	0.4

**Table 5:** Gas transport properties of modified polyimide membranes.

Membrane	Permeability, Barrer			Ideal Selectivity			Ref.
	H_2_	CO_2_	CH_4_	H_2_/CO_2_	H_2_/CH_4_	CO_2_/CH_4_	
P84/ND	6.7	1.61	0.022	4.1	310	75	This work
Matrimid/ZIF-1 (10%)	28.11	6.75	0.29	4	97	23	[51]
6FDA-TTM/Si-H (5%)	62.6	29.7	0.39	2.1	160.5	76	[52]
PI/MWCNT@GONRs (2%)	42.5	25.2	2.3	1.7	18.5	11	[53]
